# Supplementary material for: A widely distributed hydrogenase oxidises atmospheric H2 during bacterial growth
Source: ISME J. 2020 Jul 9;14(11):2649–58. doi: 10.1038/s41396-020-0713-4 (PMC7784904; doi:10.1038/s41396-020-0713-4)

**Supplementary information**

**Table S1. Oligonucleotide primers used in this study.**

| Primer | Sequence (5’ to 3’) | Tm (°C) |
| --- | --- | --- |
| *G. aurantiaca* 16S_fwd (515F) | GTG YCA GCM GCC GCG GTA A | 54 |
| *G. aurantiaca* 16S_rvs (806rB) | GGA CTA CNV GGG TWT CTA AT | 54 |
| *G. aurantiaca* HucL_fwd | TGC ATG GAC CGA AGC AAG | 62 |
| *G. aurantiaca* HucL_rvs | AAT GAG CGT GGC GTT GTG | 62 |
| *C. aggregans* 16S_fwd | CGA AAG AAC CTT ACC CGG GC | 61 |
| *C. aggregans* 16S_rvs | CGA TCT GCA CTG AGA CCA CG | 61 |
| *C. aggregans* HucL_fwd | CAT CGA GGG GAG AAA TGC GG | 61 |
| *C. aggregans* HucL_rvs | GGA AGA GCG GGT CGT AGT CT | 61 |
| *C. aggregans* HoxH_fwd | CGA TCC GAT TGA CTA CCG CG | 61 |
| *C. aggregans* HoxH_rvs | GGC GCG AAG GTT AGG TGA AA | 61 |
| *A. ferrooxidans* rpoC_fwd | GGT GCA GCA GGA TTC GTT CA | 61 |
| *A. ferrooxidans* rpoC_rvs | ACG AGG GAG GTC ATG GGA AG | 61 |
| *A. ferrooxidans* HucL_fwd | GGT TGG GCA AGT ACG TCT CG | 61 |
| *A. ferrooxidans* HucL_rvs | GCG ATC AGT TGC CGG GAT AG | 61 |
| *A. ferrooxidans* HyiB_fwd | GGA GAG CAA GAT CAT CGC CG | 61 |
| *A. ferrooxidans* HyiB_rvs | GGG AGT TCG GTG CCT TTG AG | 61 |
| *A. ferrooxidans* HyhL_fwd | CCA AAG CCG TGA GAT CAG CC | 61 |
| *A. ferrooxidans* HyhL_rvs | TGT ATC CAC TCG CCG CAG TA | 61 |

**Table S2 (xlsx): Microbial distribution and amino acid sequences of the group 2a [NiFe]-hydrogenase large subunit HucL.**

**Figure S1.** **Consumption of atmospheric H_2_ by three bacteria encoding group 2a [NiFe]-hydrogenases.** H_2_ mixing ratios of cultures of **(a)** *Gemmatimonas aurantiaca*, **(b)** *Acidithiobacillus ferrooxidans*, and **(c)** *Chloroflexus aggregans* are shown. Ratios were measured upon inoculation (blue bars), during mid-exponential growth (yellow bars), and in late stationary phase (red bars); the different sampling times between the cultures reflect their distinct growth parameters. Error bars show standard deviations of three biological replicates and statistical significance was tested using a two-way ANOVA with post-hoc Tukey’s multiple comparison (** = *p* < 0.01; *** = *p* < 0.001; **** = *p* < 0.0001).

**
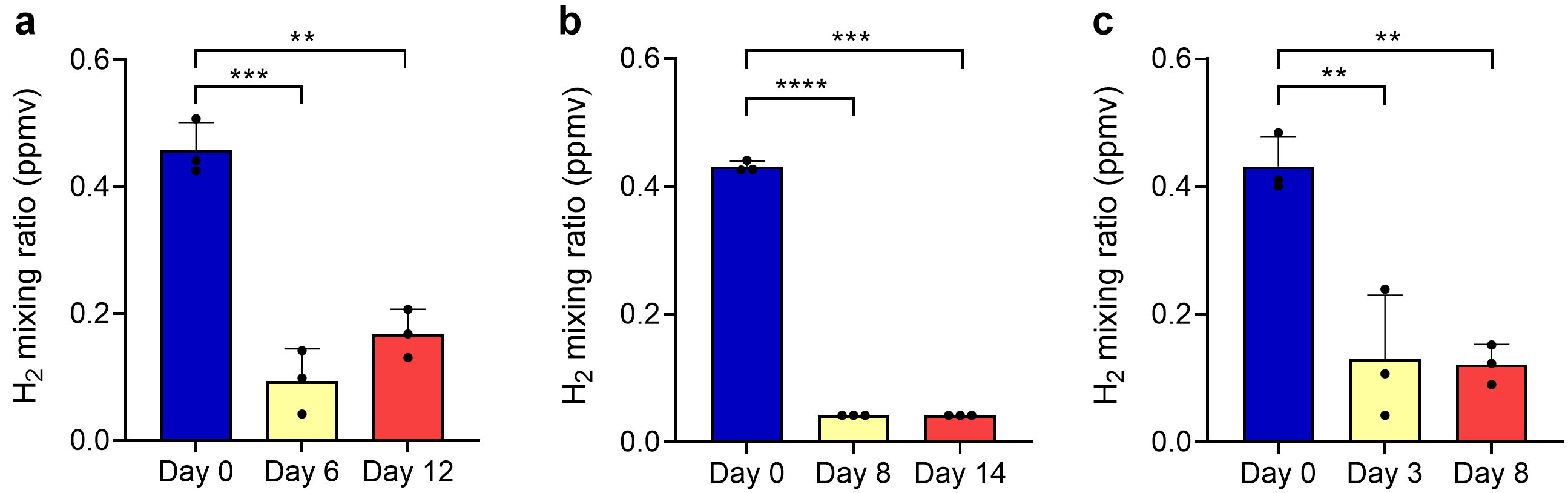
**

**Figure S2.** **Expression of other hydrogenases during growth and survival.** The normalised number of transcriptions of the large subunit genes of the **(a)** group 1e [NiFe]-hydrogenase of *Acidithiobacillus ferrooxidans* (*hyiB*, locus AFE_3286), **(b)** group 3b [NiFe]-hydrogenase of *Acidithiobacillus ferrooxidans* (*hyhL*, locus AFE_0937), and **(c)** group 3d [NiFe]-hydrogenase of *Chloroflexus aggregans* (*hoxH*, locus CAGG_2476). *Gemmatimonas aurantiaca* only encodes the group 2a hydrogenase. Results are shown for cultures harvested during exponential phase and stationary phase, in the presence of either ambient H_2_ or 10% H_2_. Error bars show standard deviations of three biological replicates (averaged from two technical duplicates) per condition. Values denoted by different letters were determined to be statistically significant based on a one-way ANOVA with post-hoc Tukey’s multiple comparison (*p* < 0.05).


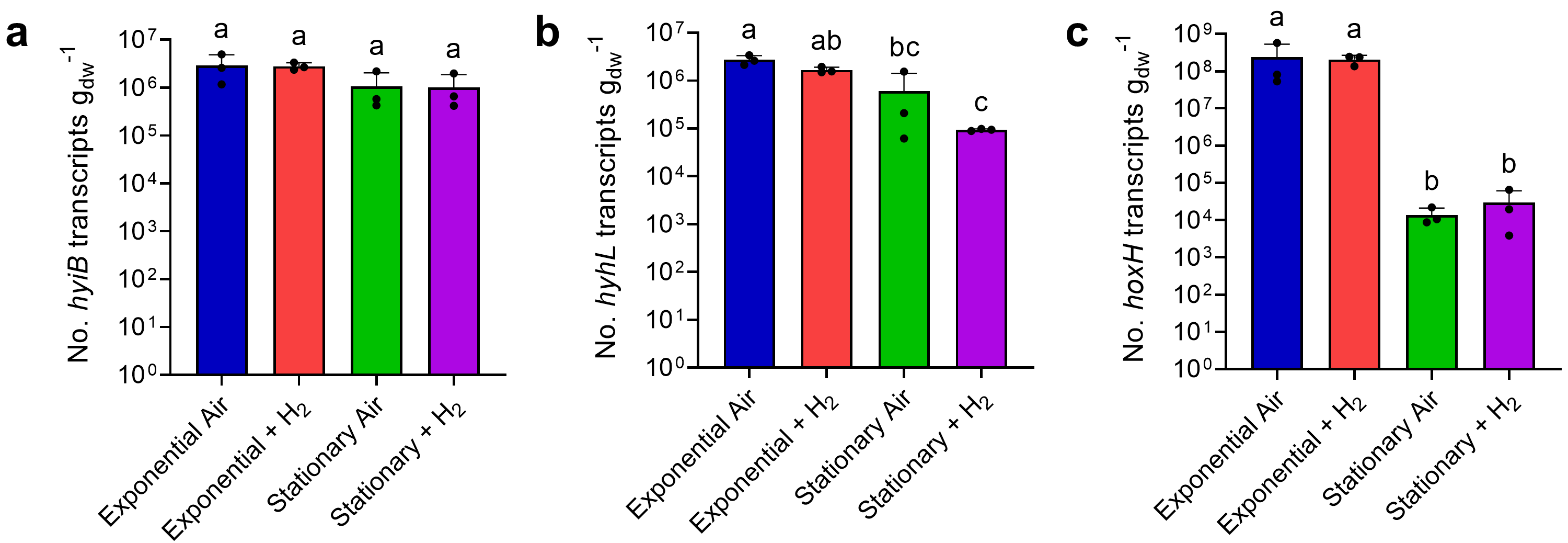


**Figure S3. Distribution of group 2a [NiFe]-hydrogenases across microbial genomes.** Results are based on the number of genomes in which the large subunit gene was detected (*hucL*) and are shown by phylum.


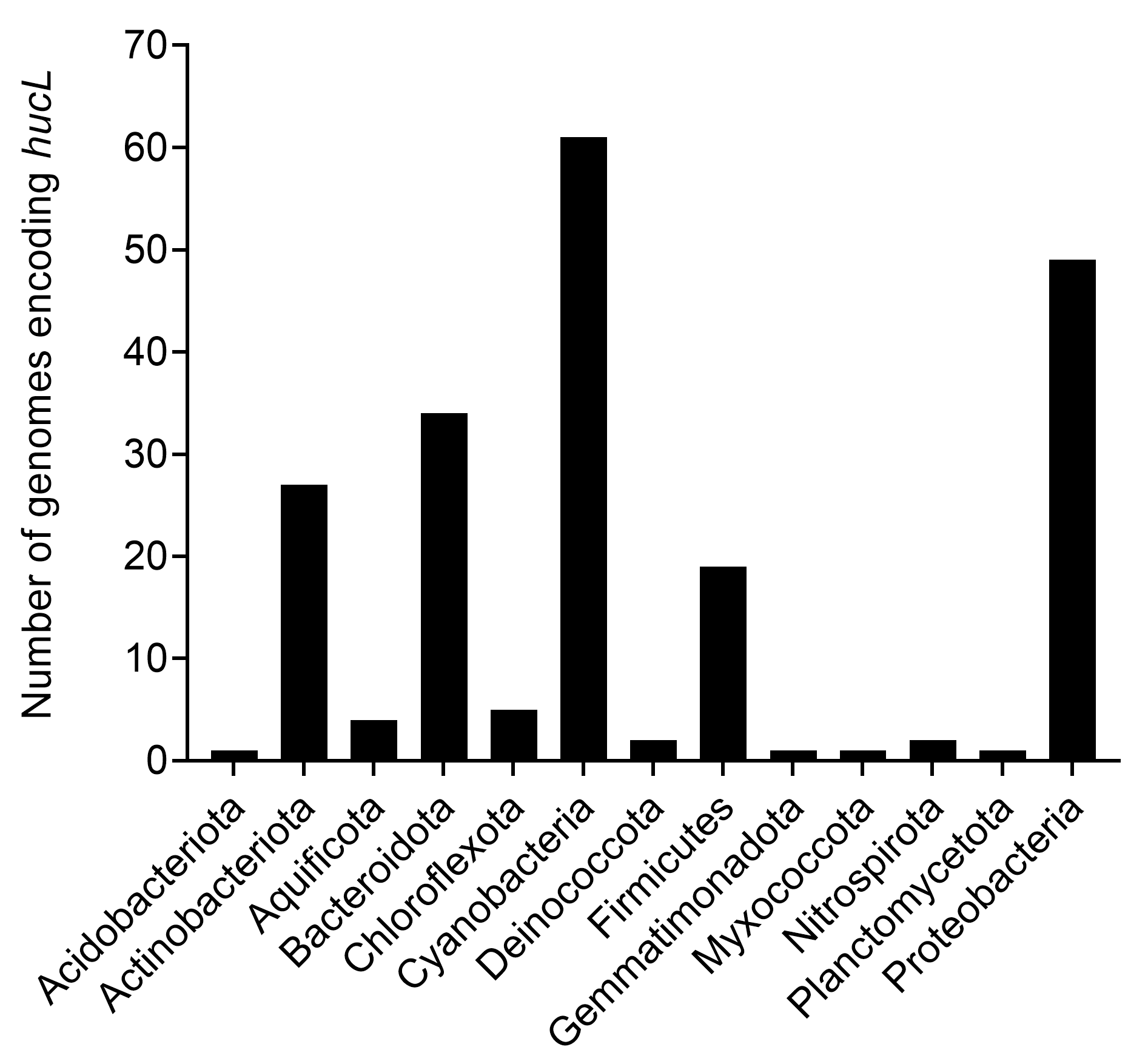


**Figure S4. Genetic organisation of group 2a [NiFe]-hydrogenases across ten phyla.** Abbreviations: HucL = hydrogenase large subunit; HucS = hydrogenase small subunit; HypABCDEF = hydrogenase maturation factors; HMP = hydrogenase maturation peptidase; NHL = NHL repeat protein; HP = conserved hypothetical protein; GmhA = putative phosphoheptose isomerase; Rieske = Rieske-like iron-sulfur protein (HucE); FAD = putative FAD-dependent oxidoreductase. Gene length is shown to scale and gene identifiers are as per the nomenclature of HydDB.


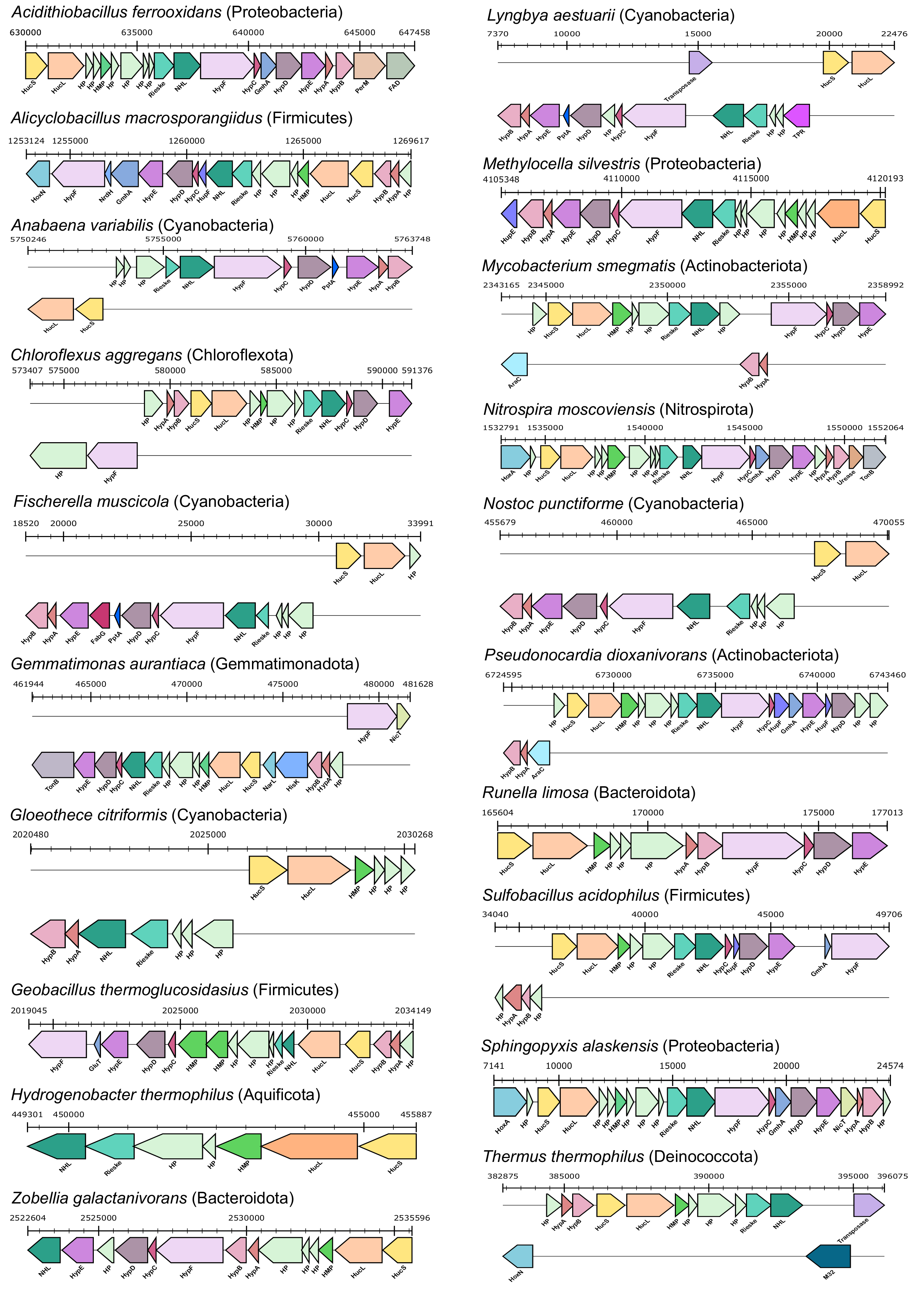

Supplement: Supplementary file 1 — Supplementary information [file 41396_2020_713_MOESM1_ESM.docx]
